# A nociceptive-nociplastic spectrum of myofascial orofacial pain: insights from neuronal ion channel studies

**DOI:** 10.3389/fncel.2024.1500427

**Published:** 2024-10-30

**Authors:** Nontawat Chuinsiri, Watcharaphol Tiskratok, Teekayu Plangkoon Jorns

**Affiliations:** ^1^Institute of Dentistry, Suranaree University of Technology, Nakhon Ratchasima, Thailand; ^2^Oral Health Centre, Suranaree University of Technology Hospital, Suranaree University of Technology, Nakhon Ratchasima, Thailand; ^3^Division of Oral Biology, Faculty of Dentistry, Khon Kaen University, Khon Kaen, Thailand

**Keywords:** myofascial pain syndrome, temporomandibular joint disorders, facial pain, ion channels, transient receptor potential channels, purinergic P2X receptors, N-methyl-D-aspartate receptors

## Abstract

Myofascial orofacial pain, traditionally viewed as a nociceptive pain condition, also exhibits characteristics consistent with nociplastic pain—pain arising from altered nociception without clear evidence of tissue damage. Evidence supporting myofascial orofacial pain as nociplastic pain includes clinical observations of central sensitisation in patients, even in the absence of visible inflammation. Sensitisation is characterised by heightened responsiveness of nociceptive neurons to normal stimuli or activation by normally subthreshold stimuli, either in the peripheral or central nervous system. It is linked to maladaptive neuroplastic changes, including increased functional potentiation and altered expression of neuronal ion channels, receptors and neurotransmitters. This mini-review presents insights from existing evidence regarding altered nociception and its relation to changes in the expression of neuronal ion channels in myofascial orofacial pain. Increased expression of transient receptor potential (TRP) vanilloid 1 channels (TRPV1), TRPV4, TRP ankyrin 1 channels (TRPA1), Piezo2 channels, P2X3 purinergic receptors, N-Methyl-D-Aspartate (NMDA) receptors and voltage-gated calcium channels in the trigeminal ganglion of rodents has been observed in association with myofascial orofacial pain. This evidence highlights the role of neuronal ion channels in the pathophysiology of myofascial orofacial pain and supports the involvement of nociplastic mechanisms.

## 1 Introduction

Neuronal ion channels regulate the flow of ions across cell membranes, which influences the transduction and conduction of pain signals. Different noxious stimuli are transduced into electrical signals via activation of specific ion channels and receptors on nociceptors, the ‘pain-sensing’ neurons. For example, noxious heat can activate transient receptor potential (TRP) vanilloid 1 channels (TRPV1) (Vandewauw et al., 2018), whereas noxious mechanical force can activate Piezo2 channels (Murthy et al., 2018). The conduction of action potentials along nerve fibres involves several subtypes of voltage-gated sodium (Na^+^) channels and voltage-gated potassium (K^+^) channels. These neuronal ion channels can become dysfunctional, leading to pathological pain conditions (Tsantoulas and McMahon, 2014; Bennett et al., 2019).

The International Association for the Study of Pain (IASP) defines three major types of pain: nociceptive, neuropathic and nociplastic. Nociceptive pain arises from non-neural tissue damage and is typically associated with injury or inflammation such as burns and cancer. It involves the activation of nociceptors, sensory neurons that respond to noxious or harmful stimuli. Neuropathic pain results from a lesion or disease of the somatosensory nervous system itself, often manifesting as electrical-like, stabbing or shooting sensations. Conditions like postherpetic neuralgia and traumatic nerve injury are examples of neuropathic pain (Cohen et al., 2021). The third type, nociplastic pain, occurs when there is altered nociception—a neural process of encoding noxious stimuli—despite no clear evidence of tissue damage or nerve injury. This pain is thought to result from central sensitisation or dysfunction in pain processing (Fitzcharles et al., 2021). Nociceptive pain is essential for survival as it alerts us of potential threats. Neuropathic and nociplastic pain, however, serve no protective function and are regarded as pathological (Cervero, 2009).

Nociplastic pain is often associated with chronic primary pain, a classification of chronic pain with an obscure aetiology that cannot be explained as a symptom of another condition (Nicholas et al., 2019). An example of chronic primary pain conditions is temporomandibular disorder (TMD) such as myofascial orofacial pain, defined as ‘pain in masticatory muscles, with or without functional impairment, not attributable to another disorder’ in the first edition of the International Classification of Orofacial Pain (Orofacial Pain Classification Committee, 2020). Myofascial orofacial pain was originally considered purely nociceptive as it is often linked to trauma and overuse of the jaws, causing inflammation in the masticatory muscles (Sharma et al., 2019). However, the persistence of myofascial orofacial pain despite the absence of physical causes or inflammation suggests the possible involvement of nociplastic mechanisms. Clinical evidence of central sensitisation, a phenomenon mostly associated with nociplastic pain, including decreased pressure pain threshold and increased mechanical temporal summation, has been observed in TMD patients (La Touche et al., 2018). Sensitisation is defined as increased responsiveness of nociceptive neurons to their normal inputs, and/or recruitment of a response to normally subthreshold inputs. Previous studies have demonstrated that sensitisation is associated with maladaptive neuroplastic changes, including functional potentiation and altered expression of neuronal ion channels, receptors and neurotransmitters at the cellular level (Latremoliere and Woolf, 2009; Ellis and Bennett, 2013). If similar changes are observed in myofascial orofacial pain, this could further support the involvement of nociplastic mechanisms in myofascial orofacial pain.

In this mini-review, we explored the current evidence regarding altered nociception and its relation to changes in the expression of neuronal ion channels in myofascial orofacial pain.

## 2 Transient receptor potential channel

Transient receptor potential channels are a diverse group of ion channels that play a critical role in various physiological processes. In trigeminal ganglion (TG) neurons, TRP channels are expressed at peripheral fibre terminals, somas (cell bodies) within the TG and central terminals in the trigeminal spinal *subnucleus caudalis* (Vc). Generally, when TRP channels are activated, they open and allow the flow of cations—such as calcium (Ca^2+^) and Na^+^—into the cell. This influx of ions alters the cell’s membrane potential and can trigger downstream signalling pathways. Peripherally, TRP channels are integral in sensing environmental stimuli such as temperature and chemicals, initiating nociceptive signalling along axons. In addition, these channels regulate the release of calcitonin gene-related peptide throughout the trigeminal nociceptive pathway. There are several subfamilies of TRP channels based on their structural and functional properties (Vandewauw et al., 2018; Citak et al., 2022; Zhang et al., 2023). Previous studies have highlighted the roles of TRPV1, TRPV4 and TRP ankyrin 1 channels (TRPA1) in myofascial orofacial pain.

Complete Freund’s adjuvant (CFA) is commonly used to induce myalgia in rodent models. Injection of CFA into the masseter muscles was shown to cause a spontaneous pain-related behaviour in wild-type mice, as evidenced by increased scores of the mouse grimace scale (MGS) on post-operative days 1 and 3. This effect of CFA was partially reduced in mutated mice with impaired PKC-mediated phosphorylation of TRPV1 in primary afferent TG neurons, suggesting that TRPV1 plays a role in mediating spontaneous pain-related behaviours in this model. The CFA-induced increase in MGS scores was transient, returning to baseline levels by post-operative day 7 for all groups (Joseph et al., 2019). In addition, pharmacological inhibition, genetic knock-out and chemical ablation of TRPV1 in the Vc were shown to attenuate CFA-induced spontaneous pain-related behaviours (Wang et al., 2017). However, knock-out and impaired phosphorylation of TRPV1, had a limited impact on CFA-induced reduction in bite force (Wang et al., 2017; Joseph et al., 2019).

Injection of CFA into the masseter muscles was reported to reduce the head withdrawal threshold (HWT) to mechanical stimulation in rats from post-operative days 3–10 (Chung et al., 2016; Bai et al., 2018). On post-operative day 3 following CFA injection, subgroup analysis showed that TRPV1 mRNA and protein levels increased in the TG of female, but not male, rats. However, in orchidectomised male rats, TRPV1 mRNA and protein levels also increased, whereas no such increase was observed in orchidectomised males receiving testosterone replacement therapy (Bai et al., 2018). Interestingly, Chung et al. (2016) and Simonic-Kocijan et al. (2013) demonstrated that TRPV1 mRNA levels were elevated in the ipsilateral TG of normal male rats on post-operative days 3 and 4, respectively, following CFA injection, coinciding with a reduction in mechanical HWT.

One study in male rats found that CFA injection into the masseter muscles reduced the mechanical HWT of the contralateral side on post-operative days 1 and 4, without affecting TRPV1 mRNA levels in the contralateral TG (Simonic-Kocijan et al., 2013).

A reduction in mechanical HWT in male rats was observed from post-operative days 1–21 following CFA injection into the masseter muscles. The HWT returned to baseline on post-operative day 28. The rat grimace scale, representing spontaneous pain-related behaviour, increased on post-operative days 1 and 3, then returned to control levels on post-operative day 7. Expression of TRPA1 mRNA levels in the TG increased from post-operative days 1–7 but returned to levels comparable to those of naïve animals on post-operative day 14 (Asgar et al., 2015).

Ligation of masseter muscle tendons in mice was demonstrated to reduce bite force from post-operative days 7–21, with bite force returning to the baseline level on post-operative day 35. Conditional knock-out of TRPV4 in TG neurons significantly mitigated the ligation-induced reduction in bite force during post-operative days 7–21. Immunohistochemistry revealed an increased percentage of TRPV4-expressing neurons in the ipsilateral TG on post-operative day 7 (Suttle et al., 2023).

## 3 Piezo2 channel

Piezo2 channels are mechanosensitive ion channels that play a pivotal role in the sensation of mechanical pain and are predominantly expressed in the peripheral terminals and somas of TG neurons (Won et al., 2017; Han et al., 2022). When activated by mechanical forces, such as tissue pressure or stretch, Piezo2 channels facilitate the influx of cations, generating electrical signals that are conducted to the central nervous system. Heightened sensitivity of Piezo2 channels can result in mechanical allodynia and hyperalgesia (Murthy et al., 2018). The exact role of Piezo2 channels in TG somas is not clear.

One study in male rats reported that CFA injection into the masseter muscles increased Piezo2 channel mRNA expression in the ipsilateral TG on post-operative day 3, coinciding with a reduction in mechanical HWT (Chung et al., 2016).

## 4 P2X purinergic receptor

The P2X purinergic receptor is activated by extracellular adenosine triphosphate (ATP), which is released during tissue injury or inflammation; the receptor is expressed in peripheral terminals, somas and central terminals of TG neurons (Goto et al., 2017; Bernier et al., 2018). Upon activation, these receptors allow the influx of cations, leading to neuronal depolarisation and the initiation of nociceptive signals. The P2X purinergic receptor activation is especially important in chronic pain, where sustained or excessive ATP release can lead to persistent receptor activation, contributing to prolonged pain sensations (Dong et al., 2022).

In male rats, unilateral injection of CFA into the masseter muscles reduced mechanical HWT bilaterally on post-operative day 4. Levels of P2X purinergic receptor subtype 3 (P2X3R) mRNA increased in the ipsilateral, but not contralateral, TG on post-operative day 4 (Tariba Knežević et al., 2016).

Daily electrical stimulation of the masseter muscles for 21 days reduced mechanical HWT, with effects beginning on post-operative days 7. An increased percentage of P2X3R-expressing neurons in the ipsilateral TG was observed on post-operative day 7 (Noma et al., 2013). In another study, four bouts of electrical stimulation-induced masseter muscle contraction reduced ipsilateral mechanical HWT 4 h after stimulation, lasting until post-operative day 7, with baseline levels returning on post-operative day 12. On the contralateral side, mechanical HWT decreased 4 h after stimulation and persisted until post-operative day 2. Real-time PCR revealed elevated P2X3R mRNA levels in the mandibular portion of the ipsilateral TG 24 h after electrical stimulation. On post-operative days 2 and 12, a higher percentage of P2X3R-expressing neurons in the ipsilateral TG was also reported (Dessem et al., 2010).

## 5 N-methyl-D-aspartate receptor

The N-Methyl-D-Aspartate (NMDA) receptor is a type of ionotropic glutamate receptors found in the trigeminal somatosensory pathway, including the peripheral terminals within orofacial tissues, the TG and the Vc. The NMDA receptor is activated by the binding of glutamate and glycine, two key neurotransmitters, and plays a crucial role in the amplification and persistence of pain. Under physiological conditions, the NMDA receptor is blocked by magnesium (Mg^2+^); prolonged and intense depolarisation removes Mg^2+^ and allows Ca^2+^ influx, enhancing nociceptive conduction and neurotransmitter release (Iwata et al., 2011; Liu et al., 2022).

Injection of nerve growth factor (NGF) into the masseter muscles reduced mechanical HWT on post-operative day 1 in male rats and from post-operative days 1–5 in female rats. The percentage of NMDA receptor subtype 2B-expressing neurons in the ipsilateral TG increased on post-operative day 3 in male rats and on post-operative days 3 and 7 in female rats, compared to the control side (Wong et al., 2014).

Ligation of masseter muscle tendons in male rats produced a persistent reduction in mechanical HWT from post-operative day 3 to week 8. In sham-operated rats, a transient reduction in mechanical HWT on post-operative day 3 was observed. Protein levels of the (un)phosphorylated NR1 subunit of the NMDA receptor increased in the superficial Vc on post-operative day 3, week 2 and week 8 in the ligation group. In the sham-operated group, a transient increase in NR1 protein levels was observed only on post-operative day 3 (Guo et al., 2010).

## 6 Voltage-gated Ca^2+^ channel

The voltage-gated Ca^2+^ (Cav) channel regulates Ca^2+^ influx in response to depolarisation and is expressed throughout TG neurons, predominantly at somas and central terminals. The major function of the Cav channels is to control the release of neurotransmitters and neuropeptides, contributing to nociception. Traditionally, dysfunction of the Cav channel is associated with neuropathic pain, and the channel is a key target in current therapies for neuropathic pain (Amrutkar et al., 2011; Guo et al., 2024).

Masseter muscle tendon ligation persistently reduced ipsilateral facial mechanical sensitivity of male rats from post-operative days 4 to 14. Messenger RNA levels of the alpha-2-delta-1 subunit of the Cav channel in the ipsilateral TG and Vc were significantly increased, compared to the sham-operated group (Lu et al., 2024).

## 7 Discussion

**Figure 1:**
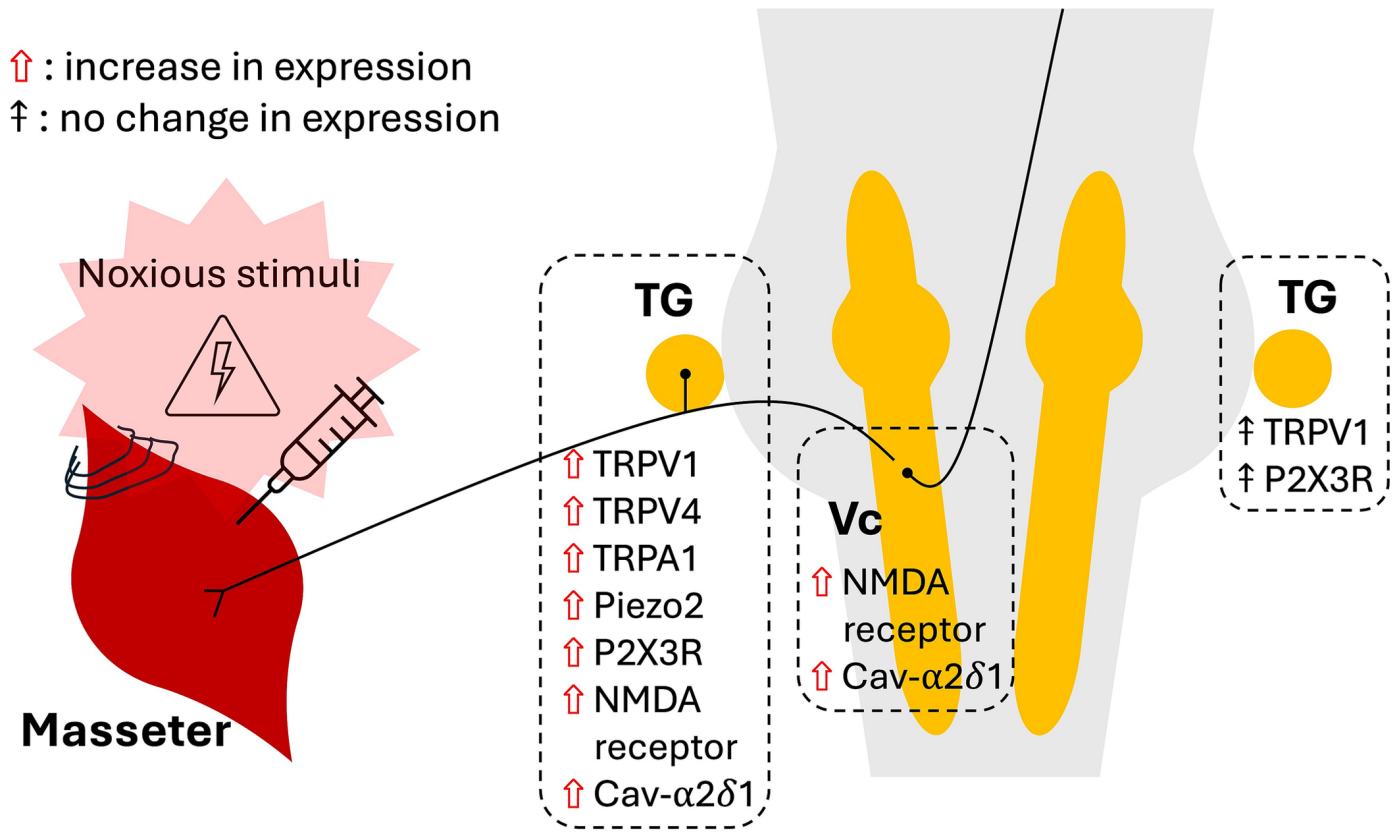
Schematic depicting the effects of noxious stimulation of the masseter muscles on the expression of ion channels in the trigeminal ganglia and *subnucleus caudalis*.

This mini-review highlights several studies examining the role of neuronal ion channels in the pathophysiology of myofascial orofacial pain. The identified models of myofascial orofacial pain are based on trauma to the masseter muscles, including direct intramuscular injection of CFA and NGF, ligation of masseter muscle tendons and electrical stimulation of the muscles. Pain-related behaviours, such as mechanical HWT and the grimace scale, were used to assess the development of orofacial pain. Upon reviewing the literature, evidence has emerged revealing that myofascial orofacial pain exhibits mechanisms of nociplastic pain: (1) changes in the expression of neuronal ion channels, (2) dependence of pain development on the proper functioning of these ion channels, and (3) bilateral pain presentation. Summaries of the current evidence are illustrated in Figure 1.

Studies indicate that the development of myofascial orofacial pain-related behaviours relies, at least partially, on the functions of TRPV1 and TRPV4 in primary afferent TG neurons (Wang et al., 2017; Joseph et al., 2019; Suttle et al., 2023). Different ion channels appeared to regulate different types of pain-related behaviours (Wang et al., 2017; Wang et al., 2018). Induction of myofascial orofacial pain has been shown to upregulate the expression of TRPV1, TRPV4, TRPA1, Piezo2 channels, P2X3R, NMDA receptors and the alpha-2-delta-1 subunit of the Cav channels in the TG (Dessem et al., 2010; Noma et al., 2013; Simonic-Kocijan et al., 2013; Wong et al., 2014; Asgar et al., 2015; Chung et al., 2016; Tariba Knežević et al., 2016; Bai et al., 2018; Suttle et al., 2023; Lu et al., 2024). These changes in the TG neurons reflect mechanisms of peripheral sensitisation and thus altered nociception. In addition, evidence of central sensitisation was demonstrated by the persistent upregulation of phosphorylated NMDA receptors in the Vc up to 8 weeks after masseter muscle tendon ligation (Guo et al., 2010). This combined molecular evidence of peripheral and central sensitisation corroborates the role of nociplastic mechanisms in myofascial orofacial pain.

Additional evidence of altered nociception is provided by studies showing that unilateral stimulation of the masseter muscles produced bilateral reduction in mechanical HWT (Dessem et al., 2010; Simonic-Kocijan et al., 2013). This bilateral manifestation of pain is also observed in trigeminal nerve injury-induced neuropathic pain, suggesting similar underlying mechanisms (Chuinsiri et al., 2021). While the exact mechanisms in myofascial orofacial pain are not fully understood, they are likely to involve spinal and/or supraspinal levels (Bułdyś et al., 2023). One possible mechanism is that TG neurons on the side ipsilateral to the stimulated muscles may cross to the contralateral side and activate central nerve terminals of contralateral TG neurons. Released neurotransmitters might also directly activate contralateral Vc neurons, which then conduct signals to the brain for processing (Samsam et al., 2001). Prolonged stimulation of contralateral TG and Vc neurons could potentially lead to alterations in the function and expression of neuronal ion channels (Ellis and Bennett, 2013). However, further studies are needed to elucidate these mechanisms in myofascial orofacial pain.

Initially, pain arising from tissue inflammation induced by CFA and ligation is likely to be nociceptive. Studies confirming changes in the expression of neuronal ion channels provide convincing evidence for a nociplastic component in myofascial orofacial pain. However, most studies reported these changes at only a single time point. To robustly demonstrate the nociplastic nature of myofascial orofacial pain, empirical evidence of persistent changes in neuronal ion channel expression, even after the resolution of the initial traumatic injury to the masticatory muscles, is crucial; such evidence is currently lacking. Future studies demonstrating persistent pain and altered expression of neuronal ion channels beyond the typical period of wound healing will be essential in addressing this unresolved question. In addition, studies investigating functional changes in conjunction with expression of neuronal ion channels are still lacking and warrant further investigation.

This literature review focused solely on studies utilising models of direct stimulation or trauma to the masseter muscles. Other studies employed models such as occlusal interference and restraint stress to induce orofacial pain-related behaviours in rodents (Zhao et al., 2015; Mo et al., 2023). However, current behavioural assays make it challenging to determine whether the pain is specifically myogenous, as similar pain-related behaviours, such as changes in mechanical HWT, can be observed in myogenous, arthrogenous, and neuropathic orofacial pain (Bi et al., 2020; Chuinsiri et al., 2021). At present, there are no behavioural assays specifically designed for myofascial orofacial pain in laboratory animals. Future research into the development of myofascial orofacial pain model based on its multifactorial aetiology and specific behavioural assays for each type of pain is encouraged.

In conclusion, the presence of pain-related behaviours in association with altered expression of neuronal ion channels following masseter muscle injury highlights the role of neuronal ion channels in the pathophysiology of myofascial orofacial pain and provides some evidence to support the involvement of nociplastic mechanisms. Further investigation is needed to elucidate the extent of these ion channels’ involvement in pain persistence and their potential as therapeutic targets.
